# A quantitative assessment of continuous versus structured methods for the detection of marine mammals and seabirds via opportunistic shipboard surveys

**DOI:** 10.1038/s41598-024-68512-6

**Published:** 2024-08-13

**Authors:** Benjamin Viola, Peter Puskic, Stuart Corney, Neville Barrett, Bronwyn Davies, Ella Clausius, Martin Jutzeler, Mary-Anne Lea

**Affiliations:** 1 Institute for Marine and Antarctic Studies, University of Tasmania, Battery Point, TAS 7004 Australia; 2 Centre for Marine Socioecology, University of Tasmania, Sandy Bay, TAS 7005 Australia; 3 Centre for Ore Deposit and Earth Sciences, School of Natural Sciences, University of Tasmania, Hobart, TAS 7001 Australia; 4 School of Natural Sciences, University of Tasmania, Hobart, TAS 7001 Australia; 5 Australian Antarctic Program Partnership, University of Tasmania, Sandy Bay, TAS 7005 Australia; 6 Australian Centre for Excellence in Antarctic Science, University of Tasmania, Sandy Bay, TAS 7005 Australia

**Keywords:** Biodiversity, Biooceanography, Community ecology, Conservation biology, Macroecology

## Abstract

Marine monitoring efforts are increasingly supported by opportunistic shipboard surveys. However, opportunistic survey methods often require adaptation to suit the vessel and the operations being conducted onboard. Whilst best-practice techniques for surveying marine wildlife on vessels of opportunity are yet to be established, testing and development of alternative methods can provide means for capturing ecological information in otherwise under-surveyed areas. Explicitly, survey methods can be improved while baseline ecological data for new regions are gathered simultaneously. Herein, we tested different survey approaches on a vessel of opportunity in a remote offshore area where little is known about the community composition of top-order marine vertebrate predators: western and south-western Tasmania, Australia. We found that continuous surveys provide greater species counts than structured “snapshot” surveys over the course of a voyage, but that structured surveys can be more practical when managing factors such as observer fatigue. Moreover, we provide a baseline dataset on the marine vertebrate community encountered in western and south-western Tasmania. This information will be critically important for industry and conservation management objectives, and is key to our understanding of the offshore ecosystem around Tasmania.

## Introduction

Quantifying interactions between marine vertebrate predators and their environment is a well-established and effective way to monitor marine ecosystems and ecosystem health^1–3^. As top-order predators, marine vertebrates such as mammals and seabirds often cover large distances, and sample multiple trophic levels when foraging, making them ideal indicator species for monitoring changes within the marine environment. Explicitly, changes in marine mammal and seabird *diversity* can indicate changes to prey fields, and changes in *abundance* can indicate that lower trophic levels within the food web are being altered^3^. Furthermore, changes in either metric can be associated with external pressures such as disease, fishing and related bycatch, pollution, and habitat degradation^2,4^.

One common method of gathering at-sea data on marine mammal and seabird species, distribution, and abundance is via shipboard surveys^5–7^. However, despite this commonality, information on at-sea survey methodology (i.e., the study of methods) is relatively scarce, particularly in the primary literature. Within what information exists, aerial surveys have been regularly compared to shipboard surveys—measuring count performance by comparing density estimates, as well as observer ability to provide species-level identification^8,9^. In contrast, comparisons between shipboard survey methods, where techniques change depending on vessel and location^10^, are especially limited. For example: Burnham et al*.*^11^ assessed biases and varying efficacy of different survey methods; Baker et al*.*^12^ made recommendations for standardised approaches relevant to voyages that have a primary focus on geology and geophysics in the United States of America; Camphuysen et al.^13^ and Tasker et al*.*^14^ made inroads towards the standardisation of sea census techniques in the United Kingdom; and Pyle^15^ did the same for West-Coast National Marine Sanctuaries in the United States of America. Camphuysen et al*.*^13^, Tasker et al*.*^14^, and Pyle^15^ all focussed on transect-based methods that are broadly applicable to projects whose primary focus is seabird or marine mammal counts. Variations of these methods are implemented globally, but most at-sea surveys for birds or mammals contemporarily occur via strip-transects or line-transects^14,16–20^. Conceptually, these transect methods are the same as their terrestrial counterparts. That is, the strip-transect involves sampling a linear area (with varying efficiency associated with strip width^11^) and extrapolating results to the total area^21,22^; whereas the line-transect requires an observer to travel in a straight line of known distance, with distances and bearings between an observation and the observer measured^23^. Well established equations are then used to derive density^21^.

Comparatively, for surveys and observations that take part on platforms of opportunity (e.g., those that are associated with industry^24^, taken from fixed platforms^10^, or on research voyages with alternate foci^25^), where methods are subject to vessel operations, there remains considerable scope to investigate and refine survey procedures, particularly with regards to estimating distribution and abundance of non-cetacean families and populations in the southern hemisphere (i.e., we acknowledge that international commitments to the protection and monitoring of whales has generated considerable methodological progress for the detection of cetaceans from vessels of opportunity^26^).

Conducting various types of research on platforms of opportunity has enormously benefitted our understanding of (and capacity to measure) baseline conditions and variability across a range of marine observing programs. In some instances, this has subsequently improved data acquisition and led to technological advancement. For instance, expendable bathymetric thermographs (XBTs) were first deployed in the 1960s, and have since generated multi-decadal long-term climate records and oceanographic data^27^. Their utilisation led to a growing community movement that recognised the importance of widespread, global data, and from that community came the Argo Program—a global array of floating devices for observing the subsurface ocean^28^. Further, in the field of hydrochemistry and hydrobiology, the implementation of continuous plankton recorders (CPRs) has generated the most extensive marine biological sampling programme in the world^29^. Moreover, citizen science initiatives such as the Secchi disk project mean that areas infrequently accessed by researchers are gaining valuable information on primary productivity through water transparency and turbidity samples^30^. The success of these programmes is testament not only to the simplicity and ease of data collection for end-users, but to the rigorous, standardised and repeatable methods.

Pertinently, marine ecologists, biological oceanographers, and marine practitioners are beginning to collect data on vessels of opportunity such that a discussion around the standardisation of techniques is required and emerging. In the northern hemisphere, aerial surveys remain a viable and widely used option for seabird and marine mammal surveys^31^. However, the northern and southern hemisphere marine vertebrate community assemblages, and their at-sea environments, are vastly different^32,33^ (for example, some species in the southern hemisphere are so similar that only detailed surveys conducted from marine vessels can accurately distinguish between them^34^). When considering methods, this ecological disparity is often not addressed, and many southern hemisphere projects lean on ocean survey protocols developed in the north. Additionally, the economic feasibility of aerial surveys in the southern hemisphere is reduced, due to lower human population density, less land/more water to fly over, and less money to do so. It is also worth noting that aerial survey protocols are not always adequate for southern hemisphere species assemblages, and that even in the northern hemisphere, aerial surveys only detect 59–77% of the abundance detected by vessel-based surveys^35^. Aerial surveys do, however, provide greater spatial coverage, which helps with species density estimates^36^.

Shipboard observations appear to provide greater species resolution^8,36^, and are a cost-effective method for gathering long-term monitoring data—particularly if associated with industries such as ecotourism or fisheries. For instance, in South Atlantic waters off South America, observations from cruise ships and tourist vessels have been found to be an effective source of data from which abundance characterisations of *Balaenopteridae* can be assessed, provided that data collection occurs in a manner befitting distance sampling analyses^26^. And for seabirds in the South Atlantic Ocean, observers on fishing vessels are collecting data that enables dietary and behavioural insights for species at-sea^37^.

Globally, the implications of at-sea ecological datasets for research are numerous and exciting, and while the field is in its infancy for marine vertebrates—where the testing of methods for a global standard ecological survey structure is underway—we can realise greater benefit by performing tests in regions that have received historically low attention. Specifically, targeting areas of historically low attention contributes to the generation of important baselines that inform ecological knowledge on at-sea species occurrence and spatial distribution^38^. This is particularly beneficial for management decisions relevant to changing environmental conditions^38^.

Acknowledging the former, this study targeted an area with limited survey effort [previously] directed towards its marine mammal and seabird community—west and south-west Tasmania, Australia. The west/south-west coast of Tasmania is subject to various commercial activities. Since the late 1960s, the oil and gas industry has undertaken several operations throughout the Sorell, and Otway basins^39^. The region is highly suitable for renewable energy interests, and thus subject to future wind-farm development plans^40^. Notably, the southern coast of Tasmania hosts a large conservation reserve that extends westward below the state: The Tasman Fracture Marine Park. This park spans 42,501 km^2^, and was proclaimed by the Australian Commonwealth Government in 2005 to protect waters around the largest global breeding colony of Shy Albatross *Thalassarche Cauta* (as per government documentation: https://parksaustralia.gov.au/marine/parks/south-east/tasman-fracture/#overview last accessed: 13/07/2023). The waters around the park are frequented by commercial fisheries including those that target Blue Grenadier *Macruronus novaezelandiae*, Southern Rock Lobster *Jasus edwardsii*, and the critically endangered Southern Bluefin Tuna *Thunnus thynnus* (Australian Fisheries Management Authority: https://www.afma.gov.au/species, last accessed: 05/05/2023). Whilst some work has touched upon the interactions between these fisheries and seals^41^, there remains little to no information on the community composition of marine top-order vertebrate predators (defined here as cetaceans, pinnipeds, and seabirds) in the region.

Hereby, this study seeks to address three key knowledge gaps: (1) Are there differences in species detectability between continuous and structured (snapshot) shipboard surveys of marine vertebrates; (2) How do continuous and structured surveys compare in terms of time/cost-effectiveness and relevance for monitoring and management purposes; and (3) Which species compose the marine vertebrate community in offshore waters from western/south-western Tasmania? Beyond these questions, this paper also aims to introduce a valuable, open-access data source that can be used for many species-specific and climate related questions.

## Results

After traversing 451.87 nautical miles, and performing 252.33 h of survey effort, we identified 45 species of marine vertebrate predator (seabirds, and marine mammals), and 11,645 individuals to species level in the survey area (survey area shown in Fig. 2—see “Methods” section. Species summaries shown in Table 1. Numbers reported exclude incidental, fish, and plastic records).

**Table 1:**
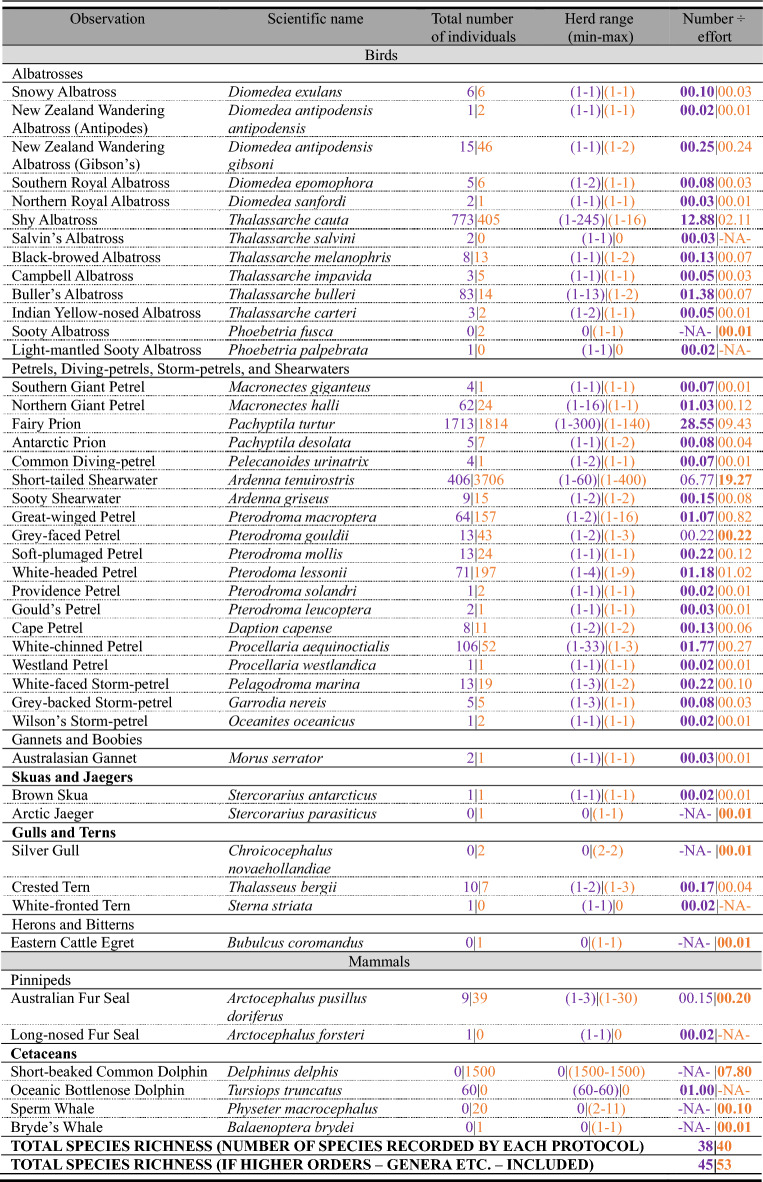
Summary of all observations (that were identifiable to species level) made on board the RV *Investigator* during the IN2023_V02 voyage for continuous and structured survey protocols.

For all columns, protocol type is reflected in the position and colour of text, separated by a pipe/vertical bar “|”: Left = Structured (purple), and Right = Continuous (orange). Higher values in the “Number ÷ effort” column are shown with bold text (structured effort = 60 h, continuous effort = 192.33 h). Species names reflect IOC World Bird List v14.1.

When assessing the number of species recorded by each survey protocol to species level identification, structured surveys detected 38 species, and continuous surveys detected 40 species (Table 1). When expanding this assessment to include higher orders such as genera (i.e., those that could not be identified to a species level), structured surveys detected 45 order/species, and continuous surveys detected 53 order/species (Table 1). These differences were significant (LRT, *p* < 0.01; Wald’s F Test, F = 318.71, *p* < 0.01, Table 2).

**Table 2:** Summary table of Wald’s F test (type II) on the full model.

Variable	F-statistic	*P*-value
Protocol	319.71	*p* < 0.01
Visibility	14.12	*p* < 0.01
Swell direction	21.50	*p* < 0.01
Swell height	5.61	*p* = 0.02
Sea-state	14.63	*p* < 0.01
Wind direction	39.91	*p* < 0.01
Wind speed	49.13	*p* < 0.01
Cloud cover	117.73	*p* < 0.01
Sea-surface temperature	0.39	*p* = 0.53
Bathymetry	427.76	*p* < 0.01
Salinity	4.14	*p* = 0.04
Fluorescence	68.03	*p* < 0.01

Whilst environmental variables are reported as part of the output, they were only included for the likelihood ratio test (see “Methods” section). Explicitly, understanding the environmental drivers of species trends is beyond the scope of this study, but is something that can be achieved with the dataset we are presenting.

**Figure 1:**
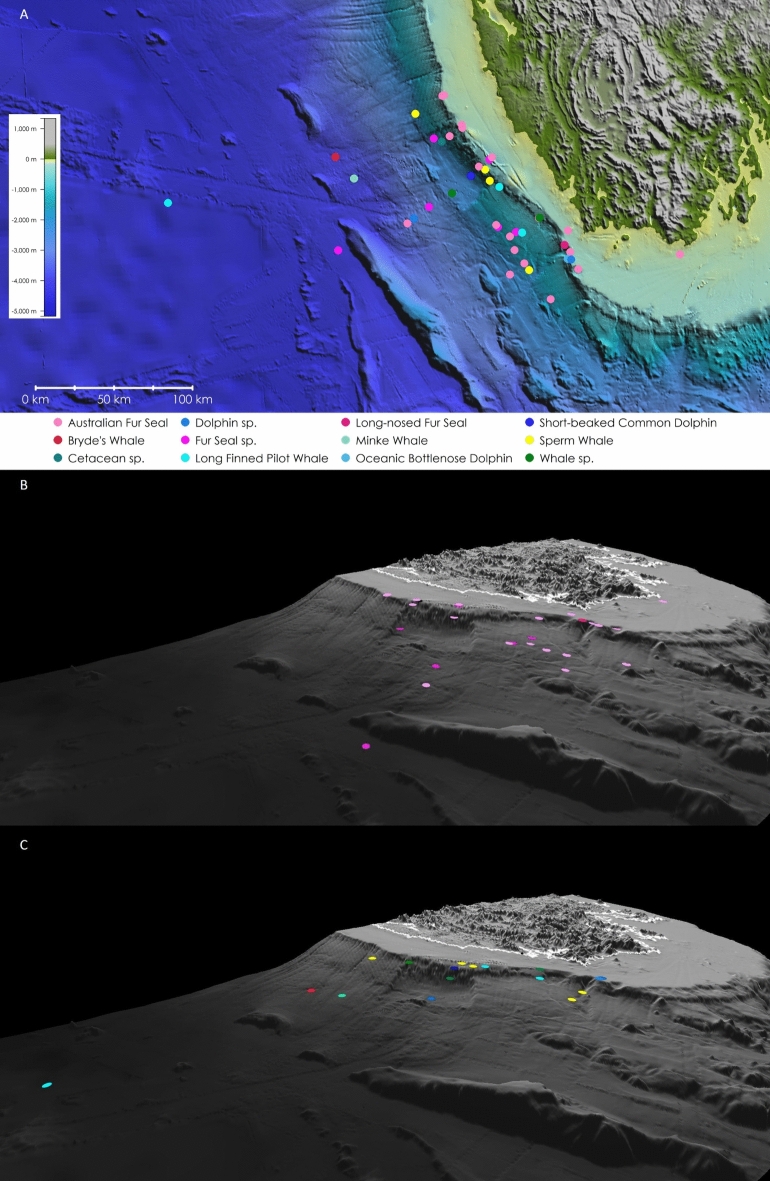
Marine mammals observed from the RV *Investigator* during the IN2023_V02 voyage. Panel A depicts all sightings—with each species described by the colours in the legend below. This colour key extends into panel B and C which depict pinniped and cetacean sightings, respectively. For panels B and C, a white boundary depicts the Tasmanian coastline. A GEBCO_2022 Bathymetric product is displayed under data. This figure was created with *GlobalMapper* (version 24.1, see: https://www.bluemarblegeo.com/global-mapper/).

Of the species detected, 33 were detected by both surveys (29 birds, 4 mammals), 5 were detected by structured surveys only (3 birds, 2 mammals), and 7 were detected by continuous surveys only (4 birds, 3 mammals) (Table 1). Twenty-six of the detected species are classified as least concern, and 19 are threatened with extinction or worse (as per the IUCN Red List of Threatened Species, https://www.iucnredlist.org/ last accessed: 17/06/2024). Whilst most species of seabird were detected via both survey protocols, marine mammals were largely detected via the continuous survey protocol (Table 1). Incidental observations recorded 22 birds and 4 marine mammals (including Long-finned Pilot Whales *Globicephala melas*, and an unidentified Minke Whale species). Marine mammals were mostly seen on and near the continental shelf between 0 and 2350 m (Fig. 1).

## Discussion

### Comparing survey methods

Here, we compare differences between two standard shipboard marine mammal and seabird survey protocols on vessels of opportunity to contrast their relative effectiveness. Whilst models accounted for the large disparity in survey effort, and despite structured surveys generally providing a greater number of detections per effort (Table 1), continuous survey protocols offer a higher likelihood of detecting a higher diversity of species than a structured survey protocol (LRT, *p* < 0.01, Wald’s F Test, F = 318.71, *p* < 0.01, Table 2). Such marginal differences observed in the total number of species detected by each protocol in this study (n = 2, Table 1) may lead readers to conclude that structured surveys are more efficient (especially when considering the amount of effort afforded to each protocol—~ 60 h for structured and ~ 190 h for continuous). However, it is important to note that when considering the duration of the voyage, the observed disparity in species richness between the two detection methods would expand over time. Intuitively, given infinite time and space, both protocols would eventually encompass all species present in a given area. Realistically however, it is more probable that continuous observations would capture every detection recorded by structured protocols, while the reverse may not hold true. Despite this, there are several additional considerations that should be made prior to the commencement of any survey:

1. Target species

As evidenced by our dataset, day-to-day variation in species occurrence, and abundance is present. Seabird abundance is known to fluctuate with weather and sea conditions, and whilst it’s implausible to predict the abundance of any given species on any given day, fluctuations in abundance can be compensated for over time provided there is repeated survey effort (where the magnitude of abundance becomes more valuable than precise abundance estimates^42^). Notwithstanding abundance, the detectability and occurrence of different species is something observers can account for and address in their study design. Pointedly, occurrence is related to seasonality and environmental drivers^43^. Surveys can therefore be temporally and spatially targeted to maximise the likelihood of encountering target species. However, when dealing with species that are rare, sparse, or inhabit subsurface environments (such as Cetacea or Pinnipedia)^32^, species detectability becomes the major consideration.

For example, in our study the intra-species variation between survey protocols was minimal for seabirds, but considerable for mammals. This is likely an artefact of the survey protocols. That is, both groups (seabirds and marine mammals) are wide-ranging predators—but the capacity for flight can enhance the detection likelihood of seabirds^44^, and therefore with increasing effort, reduces the chance of an observer ‘overlooking’ a species’ presence within a region. Conversely, the detection of marine mammals (especially individuals, i.e., those that are not part of a pod/herd) require an observer to be looking in the mammal’s direction at the exact moment that it surfaces^45^. This is easier with surface rafting species such as pinnipeds, or porpoising species such as Delphinidae, but is difficult with species such as Balaenoptera who only come to the surface for short periods of time^32,46^. Consequently, continuous surveys inherently increase the chances of encountering marine mammals, and our results reflect this. However, our incidental records capture an even greater diversity of marine mammals, suggesting that marine mammal diversity is easily under sampled—and perhaps snapshot surveys (i.e., time-restricted) are inadequate for the detection of mammals’ in lieu of continuous watch.

(2) Observers

As observers’ ability to detect species is affected (regardless of viewing conditions) by external factors such as training, experience, motivation, and fatigue^47^, we believe it is important and beneficial to design survey methods for projects on vessels of opportunity in consultation with the designated observers. It is also important to consider the accessibility of viewing platforms to observers (relevant to the vessel—e.g., are there restricted access areas that would impede sightings?), and the aims of a project piggybacking on vessels of opportunity: As the data being collected on these vessels are not the primary focus of the voyage, how can observers collect data in a way that increases output and *simultaneously* addresses the proposed research question? For instance, it has been found that tourist vessels provide a suitable platform for baleen whale observations, but that abundance can only be estimated *provided* that 12 or more vessels collect data^26^. Finally, it is good practice to consider variations in survey method, or alternate survey techniques. For example, when monitoring for cetaceans, there are a number of methods used (mark-recapture, line-transect, acoustic monitoring, etc.)—each with a distinct set of advantages and disadvantages^10^. Not all these methods are applicable on vessels of opportunity, and some are not appropriate for the monitoring of different clades of marine vertebrate, but many of these approaches can be developed, adapted, and applied to Southern Ocean studies.

For this study, it was the firm consensus of all observers that maintaining watch for all marine mammals and seabirds was less fatiguing than maintaining watch for cetaceans only (based on their subjective feelings of tiredness—rather than quantitative measurements). This was because data entry remained relatively constant when observing for all marine vertebrates, which meant that the observers felt more engaged with the task, and were actively stimulated for the watch period. Conversely, when observing for cetaceans only, there were long periods between sightings, and boredom led to fatigue. Though it was not explicitly tested within this study, future studies could pursue a similar approach by assessing/measuring observer fatigue and sighting efficiency as the primary research question.

(3) Time

Though structured surveys detected fewer species when compared to continuous surveys, structured surveys presented several benefits that should also be considered on vessels of opportunity. Firstly, structured surveys freed up observer time and allowed them to assist with other operations onboard the scientific vessel. Secondly, they allowed observers to make decisions on when to make best use of their time and perform surveys. For example, there were days when the vessel returned to the east coast to shelter from storms, and surveys were not required. This is a decision that would likely be overlooked, and not possible if continuous surveys were planned for the entire voyage. Finally, structured surveys left observers far less fatigued, and because they have a set area being surveyed, can be used to generate density estimates comparable to other surveys^10,48^.

Unfortunately, there were insufficient observers onboard to implement a paired survey approach, which involves conducting each survey protocol simultaneously. To effectively employ this approach, it would be beneficial to have a dedicated project with a primary focus on testing methods. We strongly encourage future studies to consider this if it is feasible. We further encourage future studies to measure distance to all sightings (regardless of protocol) to enable density estimates via distance sampling. In our study, distances to cetaceans were measured as per EBPC requirements, but we see no barrier as to why this couldn’t also be applied to seabirds. Understanding the environmental drivers of abundance and richness was also beyond the scope of this study, but could be assessed by future work. As an initial assessment, it appears that richness is affected by detectability (indicated by the high F-values of protocol and cloud cover in Table 2), and features associated with biological productivity (such as bathymetry and fluorescence^49^, Table 2).

**Figure 2:**
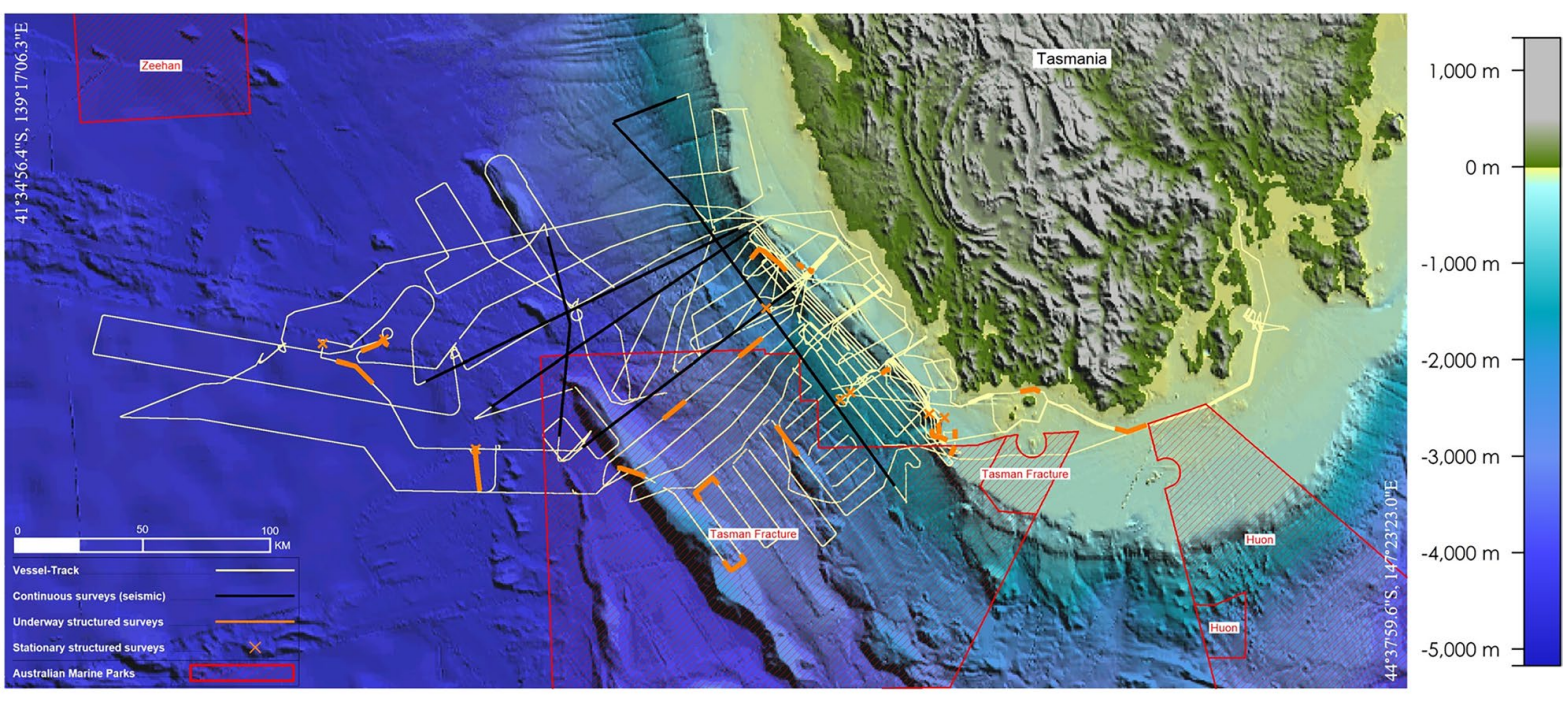
Elevation map of study area (west and south-west Tasmania) with ship tracks (4193 nautical miles) shown in cream, continuous survey effort shown in black, structured survey effort shown in orange, and marine national parks shown in red. A separate legend for bathymetry (depth, GEBCO_2022) is shown to the right of the figure. This figure was created with *GlobalMapper* (version 24.1, see: https://www.bluemarblegeo.com/global-mapper/).

Fortunately, the habitat coverage (i.e., bathymetry)was reasonably balanced between the two survey types (Fig. 2). By utilizing weighting based on effort (Table 1) and employing our statistical approach, we were still able to make some initial and meaningful comparisons—namely, with regards to our research questions, we can answer the first two such that (1) there are differences in species detectability between survey protocols, and (2) efficacy is related to purpose, and purpose needs to be defined prior to an opportunistic voyage to derive the best outcomes for research.

### Describing the seabird and marine-mammal community off West Tasmania

Beyond findings relevant to survey protocols, our study provides an important and detailed baseline capturing marine vertebrate fauna occurrence off the west and south-west coast of Tasmania. In line with our third research question, we recorded 45 species to a species level with the survey protocols described within this paper (Table 1). However, if we include incidental records (not included in this study, but part of the open access dataset available via request at https://data.csiro.au/, or by contacting the authors), an even greater number of detections (n = 47, and 70 if higher orders are included) is realised. Notably, some incidental records clearly describe species not detected by the continuous or structured survey protocols but not identified to a species level resolution (e.g., Minke Whale sp. *Balaenoptera*). In itself, this provides some reason for more survey effort in the study area as there are more species to be detected within the region. Another, arguably more compelling reason for additional survey effort, is to account for seasonality and the subsequent changes in community composition due to seasonal migrants^50^. That is, due to the operational nature of the primary project to which these opportunistic surveys were attached, dates were selected during the season known to have the lowest chance of cetacean activity for seismic reflection purposes. Ideally, to comprehensively understand the marine community ecology of western Tasmania, surveys will have to occur during months of high biological activity, when the acoustic environment is far less disturbed.

Despite surveys occurring in-between the peak seasons for Austral winter and Austral summer seabird migrants in south-east Australia (i.e., it is expected that fewer species were present compared to winter and summer^50^), of the 11,645 individuals we identified to a species level, the majority were either albatrosses or petrels (Table 1). Of these, 12 species (9 albatrosses and 3 petrels) are listed as threatened, endangered, or critically endangered (as per the IUCN Red List of Threatened Species). Additionally, there was a high number of detections for the globally vulnerable (as per the IUCN Red List of Threatened Species) Sperm Whale *Physeter macrocephalus*. Given that seabirds are the most threatened group of birds in the world, with more than one third of species at risk of extinction, and many more showing population decline^51,52^, our observations suggest the west coast of Tasmania may be an important foraging or migratory pathway for many seabird species. Hereby, it warrants further research attention to quantify the importance of the region as an ecological hotspot.

In fact, in the absence of formal pre-existing information regarding the seabird and marine mammal community off the west coast of Tasmania, we looked to citizen science initiatives such as *eBird*^53^ and *Birdata*^54^ to assess the novelty of our output. Unfortunately, when using *Birdata*, it was unclear which surveys were conducted by whom, and knowing that *Birdata* imports from *eBird* (for example, see: https://ebird.org/region/AU/post/birdlife-surveys/ last accessed: 19/06/2024), it was difficult to disentangle personal incidental records from our voyage to previous surveys. However, using *eBird*, we found that this dataset detects 18 species of seabird previously unreported in the study region—emphasising the importance of this dataset and its contribution to conservation management.

### Perspectives

With considerable interest in offshore wind-power infrastructure around the southern coasts of Australia, gathering information on marine vertebrate communities is paramount to making informed management decisions^36^. In Europe, where offshore wind-farms have been in operation for more than 20 years, aerial surveys are often conducted as a means to assess marine vertebrate associations with planned or established turbines^55,56^. Whilst aerial surveys are somewhat better at capturing high density counts of marine vertebrates at-sea (particularly seabirds), vessel-based observations provide far greater species resolution^8^—a disparity that is increasing over time due to improvements in photographic technologies, and the ability to assess digital images during, or after, a sighting^34^. Such resolution is vital to Australian marine ecosystem studies given the diversity and migration of many endangered vertebrate species persisting in Australian and Southern Ocean waters^57^.

Furthermore, high level species resolution is beneficial to long-term monitoring programs concerned with the impacts of climate change and species movement resulting from niche expansion or contraction^58^. Long-term monitoring programs provide insight towards macroecological trends not always detected at the breeding colony or site-specific level^43^. When coupled with tracking studies—particularly those that focus on sentinel species (i.e., predators that act as bioindicators for climate or ecosystems^2^)—behavioural insights that are individual, species, and sex-specific can be realised^59–62^.

Finally, high resolution species data assist with the validation (or eventual invalidation if enough surveys are performed with zero observations) of mechanisms such as Important Bird and Biodiversity Areas (IBAs)^63^. IBAs are part of a data driven *Birdlife International* programme focussed on conserving avian species, globally^64^. In Australian marine settings, a similar mechanism known as Biologically Important Areas (BIAs) are used to identify important areas for seabirds, but is also extended in this context to include marine mammals and reptiles, as well as some sharks and fishes. Biologically Important Areas are a major planning tool used by the Commonwealth of Australia to manage permitting of Commonwealth waters and in Australian Marine Parks based on species occurrence^65^, and they are currently under review (as announced the on Australian government website: https://www.dcceew.gov.au/environment/marine/marine-species/bias, last accessed: 14/07/2023). However, for many species, these BIAs are underpinned with very little quantitative data (often just expert opinion) and hence surveys such as this are particularly important for validation and improvement of species distribution maps. Our study provides validifying information pertinent to 14 of the 16 seabird species identified within Australia’s south-east marine region BIA. The two species not captured by this study are coastal, and whilst present in our incidental records, are generally not present in deep offshore waters where much of the survey effort occurred. With climate driven poleward shifts observed in the distribution of many Australian marine species, it remains unknown how or if some ecosystems will adapt^66^. Accordingly, we believe the dataset presented by this study is particularly valuable given the capacity of top-order predator data to detect bottom-up effects^3^.

### Conclusions

Our data provide an important baseline in what we hope to establish as an important long-term monitoring site, and help to inform management decisions such as those surrounding BIA assessments. Given the species richness and abundance of marine vertebrates, we believe targeted tracking studies, and surveys across different seasons will increase our understanding of the region, and we encourage anyone working within the area to build upon our existing dataset (open access dataset available via request at https://data.csiro.au/, or by contacting the authors). We consider this knowledge generation time-critical as marine top-order predators are susceptible to climate effects such as marine heat waves^67^, and Tasmania is projected to experience extreme changes in sea-surface temperature due to anthropogenic climate change^68^. Finally, our study highlights the need for careful consideration of methods prior to boarding a vessel of opportunity, and the need to consult voyage managers (crew, staff, scientists, tour-operators, or otherwise) to understand what survey protocols are possible within the bounds of their operations.

## Methods

### Study site and vessel details

All data were collected during the CSIRO Marine National Facilities voyage IN2023_V02 onboard the CSIRO Research Vessel RV *Investigator*. This voyage traversed 4193 nautical miles in waters near to the west and south-west coast of Tasmania, Australia (Fig. 2). All work occurred within the bounding box 41° 34′ 56.4″ S, 139° 17′ 06.3″ E, to 44° 37′ 59.6″ S, 147° 23′ 23.0″ E, between the 24th of March, and the 30th of April, 2023. Marine vertebrate surveys were opportunistic in the sense that geophysical surveys were the primary focus of the voyage. Vertebrate observations were made from the observation deck (28.15 m ASL) or bridge (25.5 m ASL). At both stations, observers could see to the horizon in all directions provided that environmental conditions permitted visibility. When seismic operations were underway, two 210 cubic inch airguns were towed at a depth of 6 m.

### Data collection

Marine vertebrate surveys were conducted alongside Marine Mammal Observer (MMO) duties associated with seismic operations. Observers were equipped with binoculars (10 × 42 magnification), reticle binoculars (7 × 50 mag.), DSLR (100-400 mm) or digital (80 × zoom) cameras. Underway global positioning system (GPS), and environmental data were acquired in real time from the onboard ship-sensors. There were two survey protocols: 1. Continuous (which were performed when seismic operations required MMOs on watch for the entire seismic survey period); and 2. Structured snapshots. Each protocol was conducted underway, and considerations pertaining to them, is described in detail below:

### Continuous survey protocol

As per Australian federal legislation (EPBC Act Policy Statement 2.1—*Interaction between offshore seismic exploration and whales*), one MMO was required on watch for marine mammals of interest the entire time that seismic operations were underway. As permit conditions require the designated MMO to collect only marine mammal data, it was decided between the team of four MMOs onboard that one to two MMOs would maintain MMO watch, whilst two MMOs would perform continuous surveys for marine vertebrate predators (mammals and seabirds). The description of methods herein relates to the marine vertebrate predator observers. Both observers were on watch for the entirety of the survey period, except for a staggered 30-min lunch break. This means that the observer effort for continuous surveys is double the survey hours minus 1 h when accounting for lunch (effort = (2 × time)—1). Continuous surveys began at first light, and continued until sunset, or until the seismic line was completed (pre-sunset). Typically, these surveys lasted 8 or 9 h. Notably, seismic operations were occasionally delayed or terminated due to cetacean encounters. When performing continuous surveys, observers were not constrained in where or how far they could look. However, they remained in constant communication to notify one-another of observed animals in an attempt to avoid double counts of individuals.

### Structured survey protocol

Structured surveys occurred when seismic operations were not underway, and followed a similar approach to other studies with similar species assemblages^69^. For these surveys, observers did not have a say on where the ship was headed—so ship activity ranged from stationary (when coring operations were underway), to steaming (when in transit, or when dredging operations were underway). During these surveys, each observer watched a 90-degree section over the bow of the ship. One observer would watch the starboard side, and the other would watch the port side. Animals were only recorded if they were seen within a 1 km radius of the observer (essentially, this method used a 2-km strip transect with 1-km either side of the ship, and distances were calculated with the use of reticle binoculars). This limit was chosen as large swells could obstruct the horizon for observers/obstruct animals behind waves, and the observers wanted to be able to maintain visuals on individual animals to avoid double counts (notably, this hindrance exists within the continuous protocol, but as there are no visual constraints on distance for continuous surveys, observers could maintain watch on an individual beyond high swell until it reappeared). Explicitly, observers would notify one another when an animal was passing by their 90-degree section and moving into the other observers’ section. For example, if observer 1 spotted a Shy Albatross on the starboard side traversing across the bow towards the port side of the ship, they would notify observer 2 and let them know to avoid double count of that individual.

Where possible, these surveys occurred within the same daylight period, three times a day: 0800–0900, 1200–1300, and 1600–1700 AEST. Notably, daylight savings ended during this voyage. Survey hours were subsequently adjusted to keep daylight hours biologically consistent between surveys, and effort was calculated as double the recorded time (2 observers, so effort = 2 × time).

### Accounting for shipboard followers

For each of the survey methods, both observers tried to account for shipboard followers (i.e., seabirds that return to, or follow a vessel during survey periods) as much as possible. This was done by maintaining visuals on circling birds, but also by noting differences in individual plumages or moult patterns. If an individual with no distinguishing features left the 1 km survey radius (during structured surveys) and later returned, it was likely re-counted.

Given the survey protocol for shipboard followers remained constant, the proportion of followers per species remains constant between surveys and comparisons in abundance are proportionate. That said, based on our observations of the birds whose plumage was unique, very few individuals return after leaving the one km radius.

### Incidental observations

Species observed outside of survey protocols were occasionally reported as incidental observations. These observations tended to be collected for species that are known to be less common, data-deficient, difficult to observe, or simply charismatic. They were not included in this analysis but are mentioned in the discussion.

### Analyses and data handling

All data analyses and handling occurred in Google Sheets (Google LLC. (2018)), Microsoft Excel (Microsoft Corporation. (2018). *Microsoft Excel*. Retrieved from https://office.microsoft.com/excel), GlobalMapper (version 24.1, see: https://www.bluemarblegeo.com/global-mapper/), and the R statistical environment (version 4.3.0,^70^). Explicitly, data were collected in a shared Google Sheet, and later downloaded to Microsoft Excel where they were screened for typographical errors. Once cleaned, the data were saved in a .csv file and imported to GlobalMapper and RStudio (version 2023.03.1 + 446) for summary outputs and analyses. Statistical outputs were determined to be significant if p-values were less than 0.01 (*p* < 0.01).

To test whether there were statistical differences between species detectability over time with different shipboard survey approaches, we performed two generalised linear models^71^, and a likelihood ratio test^72^. Each of the generalised linear models (GLMs) included the underway environmental data for each observation as explanatory variables. Environmental data included categorical measures of visibility, cloud cover, swell direction, and sea-state. They also included quantitative measurements (via shipboard sensors) of swell height (m), wind direction (°), wind speed (knots), sea-surface temperature (°C), bathymetry (m), salinity (ppm), and fluorescence (µg/L) (most of these variables are included in the final product, and all are available via the aforementioned CSIRO data portal).

The first model included an additional explanatory variable of survey protocol (weighted for effort), whilst the second model omitted this variable. All variables were tested for correlation in R using the *ggcorr* function within the “GGally” package^73^, and those with a value higher than 0.6 were removed. The response variable for both models was species richness per survey. To account for overdispersion, both models were fit with a Quasi-Poisson distribution^74^. Next, we employed a likelihood ratio test to compare models with and without the survey protocol as an explanatory variable to see if there was a significant difference in total species richness observed between the two survey methods after accounting for environmental variables. Explicitly, the likelihood ratio test tested the hypothesis that the full model (i.e., the model that included survey protocol as an explanatory variable) is a better fit to the data than the reduced model (i.e., the model that excluded survey protocol as an explanatory variable) (Eq. 1^72^).

1 $$\lambda_{{\text{LR }}} = - 2\ln \left[ {\frac{{\sup_{{\theta \epsilon \Theta_{0} }} {\mathcal{L}}\left( \theta \right)}}{{\sup_{\theta \epsilon \Theta } {\mathcal{L}}\left( \theta \right)}}} \right]$$

Here, $\lambda_{{{\text{LR}} }}$ is the likelihood ratio, where the parameter *θ* in a specified subset Θ_0_ of parameter space Θ, defines a null hypothesis where the alternate is that *θ* is in the complement of Θ_0_ in Θ. ${\mathcal{L}}\left( \theta \right)$ refers to the maximum likelihood function, and sup refers to the supremum. Thus, if the p-value was significant (*p* < 0.01), we concluded that survey protocol explained the variation in species richness after accounting for environmental variation.

To support the likelihood ratio test, we also performed a type II Wald’s F test^75^. Specifically, this test was used to assess the significance of individual regression coefficients in the context of our full model. Whilst the outcome of this test can lead to a similar conclusion as the likelihood ratio test, it is slightly more informative, and a well-established method for assessing GLM outputs where the response variable is Poisson in nature^76,77^.

### Ethics approvals

Species distribution data were collected to supplement an *Our Marine Parks 3* grant funded by the Australian Department of Climate Change, Energy, the Environment, and Water. Observational data were collected under University of Tasmania (UTAS) ethics permit #27523 and adhered to ARRIVE guidelines as per UTAS animal ethics requirements. The observers involved within the study are authors of this work (namely, Benjamin Viola and Peter Puskic). All work related to the IN2023_V02 Marine National Facility *RV Investigator* voyage occurred under The Australian Marine Park Activity Permit PA2022-00155-1 and Agreement PA2020-00041.

## Data Availability

The datasets generated and analysed during the current study are available in the CSIRO Data Access Portal, https://data.csiro.au/.
